# Non-destructive prediction of isoflavone and starch by hyperspectral imaging and deep learning in Puerariae Thomsonii Radix

**DOI:** 10.3389/fpls.2023.1271320

**Published:** 2023-10-25

**Authors:** Huiqiang Hu, Tingting Wang, Yunpeng Wei, Zhenyu Xu, Shiyu Cao, Ling Fu, Huaxing Xu, Xiaobo Mao, Luqi Huang

**Affiliations:** ^1^ School of Electrical and Information Engineering, Zhengzhou University, Zhengzhou, China; ^2^ Research Center for Intelligent Science and Engineering Technology of Traditional Chinese Medicine, Zhengzhou University, Zhengzhou, Henan, China; ^3^ School of Pharmaceutical Sciences, Zhengzhou University, Zhengzhou, China; ^4^ State Key Laboratory for Quality Ensurance and Sustainable Use of Dao-di Herbs, National Resource Center for Chinese Materia Medica, China Academy of Chinese Medical Sciences, Beijing, China

**Keywords:** Puerariae Thomsonii Radix, isoflavones and starch content, hyperspectral imaging, deep learning, one-dimensional convolutional neural network

## Abstract

Accurate assessment of isoflavone and starch content in Puerariae Thomsonii Radix (PTR) is crucial for ensuring its quality. However, conventional measurement methods often suffer from time-consuming and labor-intensive procedures. In this study, we propose an innovative and efficient approach that harnesses hyperspectral imaging (HSI) technology and deep learning (DL) to predict the content of isoflavones (puerarin, puerarin apioside, daidzin, daidzein) and starch in PTR. Specifically, we develop a one-dimensional convolutional neural network (1DCNN) model and compare its predictive performance with traditional methods, including partial least squares regression (PLSR), support vector regression (SVR), and CatBoost. To optimize the prediction process, we employ various spectral preprocessing techniques and wavelength selection algorithms. Experimental results unequivocally demonstrate the superior performance of the DL model, achieving exceptional performance with mean coefficient of determination (R^2^) values surpassing 0.9 for all components. This research underscores the potential of integrating HSI technology with DL methods, thereby establishing the feasibility of HSI as an efficient and non-destructive tool for predicting the content of isoflavones and starch in PTR. Moreover, this methodology holds great promise for enhancing efficiency in quality control within the food industry.

## 1 Introduction

Puerariae Thomsonii Radix (PTR), a perennial plant with a long history of use in Chinese medicine, has gained increasing popularity due to its well-documented therapeutic effects (Zhou et al., 2014). It is valued for its remarkable nutritional and bioactive profiles, offering a wide range of health benefits (Wagle et al., 2019; Lai et al., 2020). Rich in phytochemicals, PTR is particularly abundant in isoflavones, which serve as the primary bioactive compounds responsible for its diverse pharmacological activities (Xu et al., 2016). Notably, extensive research has focused on the presence of isoflavones in PTR, including puerarin, puerarin apioside, daidzin, and daidzein (Li et al., 2022a), highlighting their anti-inflammatory, antioxidant, and anti-cancer properties (Chen et al., 2017). Furthermore, these compounds have been associated with positive effects on cardiovascular health, reducing the risk of heart disease and stroke (Wang S. et al., 2023).

Alongside its medicinal value, PTR is extensively acknowledged as a nutritious food, providing essential nutrients such as starch, protein, and fiber that are integral to maintaining a balanced and healthy diet (Liang et al., 2017). Remarkably, PTR is particularly rich in starch, serving as an excellent source of energy and assisting in the regulation of healthy blood glucose levels (Liu et al., 2021). Furthermore, PTR boasts a significant dietary fiber content, which promotes digestive health, prevents constipation, and reduces the risk of colon cancer (Fu et al., 2023). Given its exceptional nutritional and therapeutic properties, PTR has garnered popularity as a sought-after ingredient in natural remedies and dietary supplements, offering a diverse array of health benefits (Zeng et al., 2019).

With the continuous improvement of living standards in the modern era, consumer concerns regarding food quality have gained significant prominence. Safeguarding the commercial value of PTR necessitates a focus on controlling the content of its bioactive compounds to ensure quality attributes (Li Q. et al., 2022; Zhang Y. et al., 2023). To facilitate quality control and assurance for commercial applications, it is essential to accurately predict the bioactive compound content of PTR. Traditional well-known chemical and physical strategies such as high-performance liquid chromatography (HPLC) (Niu et al., 2012), mass spectrometry (MS) (Liu et al., 2021; Shang et al., 2021), and spectrophotometry (Wong et al., 2015; Reddy et al., 2017), have been utilized for this purpose. However, these techniques come with inherent limitations, including time consumption, expensive equipment requirements, sample destruction, and the use of toxic reagents. Despite their high accuracy and sensitivity, these drawbacks have spurred the exploration of alternative methods that are faster, non-destructive, and cost-effective. Thus, there is a pressing need for a rapid, efficient, and non-destructive approach to ensure the quality of PTR.

Near-Infrared Spectroscopy (NIR) and Infrared Spectroscopy (IR) techniques offer notable advantages in substance analysis (Roggo et al., 2005; Tsuchikawa and Kobori, 2015), including non-destructive measurements and heightened sensitivity to trace components (Ozaki, 2012). However, their complexity requires specialized expertise for data interpretation, and their applicability is often limited to specific sample types (Ozaki, 2021). Additionally, these techniques may not fully capture the internal characteristics of the substances being examined. In certain situations, the acquired spectral information may be insufficient or inadequate to represent the entirety of the sample (Ozaki et al., 2021). Hyperspectral imaging (HSI) is an advanced analytical technique that combines spectroscopy and imaging to analyze the chemical and physical properties of a sample (Femenias et al., 2021). With its rapid analysis time, high spatial resolution, and the ability to simultaneously analyze multiple components, HSI has become an essential tool in the food industry for chemical property detection and quality control (He et al., 2023). By combining the rich spectral information with the capabilities of machine learning algorithms, we can effectively identify food adulteration, assess quality, and predict of component content (Kang et al., 2022; Teixido-Orries et al., 2023). However, the abundance of spectral bands in hyperspectral data poses challenges that require attention. Traditional machine learning models often rely on extensive feature engineering and selection to optimize their performance, which limits their practicality and effectiveness (Chen et al., 2014; Saha and Manickavasagan, 2021; Zhang et al., 2022a; Zhang L. et al., 2023).

Deep learning (DL) has gained tremendous popularity in recent years, driven by its remarkable ability to tackle complex problems (Mishra and Passos, 2021). aging, DL models can effectively exploit the extensive spectral information embedded within the data, leading to improved precision and resolution in regression and prediction tasks (Wang et al., 2021). For instance, Mansuri et al. (2022) employed Vis-NIR HSI to detect fungal contamination in maize kernels and developed partial least squares discriminant analysis (PLS-DA), artificial neural network (ANN), and 1DCNN models. Notably, the 1DCNN model outperformed the other methods, demonstrating superior detection accuracy (Mansuri et al., 2022). Similarly, Li et al. (2023) proposed a CNN model utilizing HSI to accurately identify adulteration in Atlantic salmon (Li et al., 2023). Cai et al. (2023) leveraged HSI and deep fusion learning approaches to determine the geographical origins of Radix Paeoniae Alba (Cai et al., 2023). Furthermore, Zhou et al. (2023) predicted lead content in oilseed rape leaves by combining fluorescence HSI with DL techniques (Zhou et al., 2023). In another study, Zeng et al. (2022) merged HSI and low-field nuclear magnetic resonance with DL to rapidly and non-destructively detect moisture content in salted sea cucumbers (Zeng et al., 2022). Additionally, Soni et al. (2021) quantified Clostridium sporogenes spores in food using HSI and compared the performance of 1DCNN and random forest models. The findings underscored the significant potential of DL, with the CNN exhibiting superior performance over the random forest model (Soni et al., 2021). These studies have substantiated the feasibility and superiority of employing DL models in conjunction with HSI for food analysis and safety inspection, surpassing traditional machine learning methods such as PLSR and RF.

As mentioned above, the combination of HSI and DL algorithms have shown significant potential for application in the field of food analysis in recent years. In this context, the main objectives of this study are as follows: (1) Explore the potential of HSI and data analysis methods to predict the levels of puerarin, puerarin apioside, daidzin, daidzein, and starch in PTR. (2) Following the conventional modeling process, compare the performance of typically used traditional machine learning prediction algorithms, with various spectral preprocessing techniques and wavelength selection methods. (3) Develop a 1DCNN model and establish its advantage by comparing its predictive performance with commonly used prediction algorithms. By addressing these objectives, this study aims to advance the accurate prediction of compounds in PTR using HSI technology and data analysis methods, providing valuable insights for quality evaluation and control in the food industry.

## 2 Materials and methods

### 2.1 Sample preparation

This study focused on kudzu (*Pueraria thomsonii*) sourced from Jiangxi Province, China. The kudzu plants were harvested during their second year of growth, and the root tuber epidermis was carefully removed using ultrasound-assisted washing. The PTR samples were then subjected to natural drying. A total of 1000 g of PTR was collected from the specified geographical origin, and 10 g of PTR were packed together to form one sample for hyperspectral image acquisition. In total, 100 samples were collected for analysis. During hyperspectral image acquisition, the PTR samples were individually placed on a black plate. Subsequently, the samples were freeze-dried and finely ground into powders to facilitate the analysis of puerarin, puerarin apioside, daidzin, daidzein, and starch.

### 2.2 Hyperspectral imaging system acquisition

The data collection process in this study involved the use of a hyperspectral imaging system equipped with two lenses: one for capturing visible light and the other for short-wave/long-wave near-infrared components (HySpex VNIR1800/HySpex SWIR 384, Norsk Elektro Optikk, Oslo, Norway). Specifically, our focus for the experiment was on the wavelength range of 948.72 − 2512.97 nm in the SWIR region, which comprised a total of 288 spectral bands. To ensure proper illumination of the samples, we employed two 150 W bromine-tungsten lamps (H-LAM, Norsk Elektro Optikk, Oslo, Norway) as the light source. The integration time for the SWIR lenses was set at 3500 *µ*s. Throughout the data acquisition process, the samples were positioned on a conveyor belt moving at a constant speed of 2.0 mm/s, while maintaining a distance of 28 cm between the samples and the lenses, the dimensions of the black plate used to place the sample is 100 cm× 45 cm.

### 2.3 Data correcting

During the process of acquiring hyperspectral images, non-uniformity in the intensity of light and interference from dark currents can lead to uneven output images (Jia et al., 2020). This can be a negative impact on subsequent data analysis. To address this, it is critical to calculate the relative reflectance using both dark and white reference images. The corresponding correction method is calculated as the following equation.


(1) $$I_{new}=\frac{I_{raw}-I_{dark}}{I_{white}-I_{dark}}$$

The corrected image is denoted by *I_new_*, and it is obtained by applying a correction method to the original hyperspectral image *I_raw_*, taking into account the dark reference image *I_dark_* and the white reference image *I_white_*.

### 2.4 Measurement of total puerarin, puerarin apioside, daidzin, daidzein, and starch

#### 2.4.1 Chemicals

Methanol (chromatography grade) and acetonitrile (chromatography grade) were obtained from Tianjin Siyou Fine Chemical Co., Ltd. Methanol (analytical grade) and formic acid (analytical grade) were obtained from Tianjin Zhiyuan Chemical Reagent Co., Ltd. Ultra-pure water was prepared in the laboratory using a Mili-Q Advantage A10 system from Merck KGaA (MA, USA). The standards used in this study included puerarin (lot number 110752-201816), daidzin (111738-201904), daidzein (111502-202003), which were purchased from the China Institute for Food and Drug Control. Furthermore, puerarin apioside (lot number 103654-50-8) was purchased from Chengdu Plantmark Pure Biotechnology Co., Ltd. All reagents were of analytical grade and were used without further purification.

#### 2.4.2 Preparation of standard solution

Four distinct compounds, namely puerarin, puerarin apioside, daidzin, and daidzein, were each dissolved in methanol to prepare standard solutions with a 20 mg amount of each compound. The solutions were made up to a final volume of 10 mL in volumetric flasks to obtain the stock solutions for each compound. To prepare a mixed standard solution, suitable volumes of the stock solutions were combined and diluted with methanol to produce a concentration gradient. Standard curves were created for each compound using the appropriate volumes of the standard solutions. This rigorous method was employed to guarantee precise and dependable measurements of the compounds in subsequent experiments. The resulting mixed standard solution and individual standard curves will be used to determine the concentration of each compound in samples.

#### 2.4.3 Isoflavones extraction in Puerariae Thomsonii Radix

Firstly, 1.0 g of the powder, which had passed through a sieve (50 mesh), was mixed with 50 mL of the 50% methanol solution. Ultrasonic extraction was then performed on the mixture at room temperature for 30 mins. The resulting mixture was cooled, and its weight was measured. Any weight loss was compensated for by adding more 50% methanol solution to the mixture. After shaking, the mixture was filtered, and the filtrate was collected for further analysis. To prepare the filtrate for injection, it was filtered through a 0.22 *µ*m microfiltration membrane and then injected into a sample bottle using an automatic sampler.

#### 2.4.4 HPLC system for isoflavones analysis

The HPLC K2025 liquid chromatography system was composed of a Binary pump, Autosampler, Column oven, and UV-VIS detector. For every analysis of the samples, a YMC-Pack Pro C18 reverse phase column was utilized with a mobile phase consisting of an aqueous solution containing 0.01% formic acid and acetonitrile. Detection was conducted at a wavelength of 250 nm, with the column temperature maintained at 30 °C, a flow rate of 1 mL/min, and an injection volume of 10L.

#### 2.4.5 Quantification of starch

A sample of PTR weighing precisely 1.0 g, which had been passed through a sieve (50 mesh), was added to 10 mL of distilled water. The resulting mixture was homogenized in a blender, filtered, and the filtrate was subjected to centrifugation at 4500 rpm for 15 mins. The supernatant was then discarded, and the resulting precipitate was dried in a 70 °C oven until a constant weight was achieved. The mass of the precipitate was recorded for the calculation of starch content using the following formula.


(2) $$W=\frac{m_{1}}{m_{2}}\times 100\%$$

Where, W is starch content, *m*_1_ is the mass of the dried precipitate, and *m*_2_ is sample mass.

### 2.5 Modeling

#### 2.5.1 Spectral preprocessing

Preprocessing plays a crucial role in hyperspectral data analysis as it helps eliminate noise, correct instrument artifacts, and enhance the signal-to-noise ratio for further analysis. In this study, we evaluated several well-established preprocessing methods, including the standard normal variate (SNV), multiplicative scatter correction (MSC), Savitzky-Golay smoothing (SG), as well as the first derivative (FD) and second derivative (SD) methods. The goal was to identify the most effective preprocessing technique that would yield the most accurate predictive models.

#### 2.5.2 Effective wavelength selection

In the analysis of hyperspectral data, the selection of appropriate spectral bands is of paramount importance for obtaining meaningful information and reducing data redundancy. Given the hundreds or thousands of spectral bands, noise and irrelevant spectral information are common, which can lead to inaccurate results or high computational costs in further data analysis. Effective band selection aims to improve the accuracy and efficiency of the analysis by reducing the dimensionality of the data while preserving the most informative spectral features. Furthermore, the selection of relevant bands can facilitate the interpretation of the data and provide insights into the underlying physical and chemical processes. Hence, it is a critical step in hyperspectral data analysis and has received significant attention in the literature.

**Successive projections algorithm (SPA):** The SPA operates by computing the coefficient of determination between each spectral band and the target variable, and subsequently selecting the feature with the highest absolute correlation in the initial iteration (Zhang et al., 2017). During each subsequent iteration, SPA eliminates the spectral band that has the least impact on the coefficient of determination of the remaining spectral bands until the desired number of features is achieved. In this manner, SPA effectively identifies a subset of the most relevant and informative spectral bands, while preserving the effective wavelengths that capture the most significant spectral features.

**Competitive adaptive reweighted sampling (CARS):** CARS is a potent algorithm that facilitates wavelength selection in HSI. Its underlying principle involves competitively sampling spectral bands iteratively. Every wavelength is assigned a weight that corresponds to its significance in the classification task (Li et al., 2009). CARS adaptively reweighs wavelengths during each iteration based on their discriminative power, and it ultimately selects the most informative subset of wavelengths.

**Uninformative variable elimination (UVE):** This algorithm achieved by measuring the relevance of each wavelength to the final task using statistical methods such as mutual information, coefficient of determination, or variance analysis. Wavelengths that exhibit low significance are subsequently removed, resulting in only informative wavelengths remaining for subsequent analysis (Cai et al., 2008).

#### 2.5.3 Conventional data analysis approaches

**Support vector regression (SVR):** SVR is a powerful machine learning algorithm that is extensively used for regression tasks. The fundamental principle of SVR is to construct a hyperplane in a high-dimensional feature space that maximizes the margin between the training data points and the hyperplane, facilitating accurate regression (Dhiman et al., 2019).

To prevent overfitting and improve generalization, SVR incorporates a regularization parameter that balances the trade-off between maximizing the margin and minimizing the training error. By adjusting the regularization parameter, SVR can effectively handle overfitting and generalize well to new unseen data, providing robust and accurate regression results.

**Partial least squares regression (PLSR):** In the realm of HSI, PLSR is a frequently employed multivariate statistical technique for predicting the concentrations of chemical components in a sample. Its primary objective is to reveal the underlying relationship between the predictor variables (spectral features) and the response variable (chemical composition content).

PLSR achieves this by decomposing the predictor variable set into a reduced number of latent variables that capture the variance in the spectral data. It then performs regression analysis between these latent variables and the response variable. The latent variables are carefully selected to maximize the covariance between the predictor and response variables, thereby ensuring that they capture the critical information and relationships between the two sets (Li et al., 2022b).

**CatBoost:** Through the use of ordered boosting, random permutations, and gradient-based optimization, CatBoost is able to deliver cutting-edge performance across a range of domains. This versatile machine learning framework boasts a crucial advantage in its ability to effectively manage categorical variables with high cardinality and handle missing data, making it a valuable tool for real-world datasets (Dorogush et al., 2018). When combined with HSI, it offers an effective approach for accurately predicting the content of various sample components, particularly in the food industry, such as fat, protein, and moisture (Zou et al., 2023).

#### 2.5.4 Deep learning approaches

One-dimensional convolutional neural networks (1DCNN) are becoming increasingly popular in HSI due to their remarkable ability to accurately predict the concentrations of chemical components in a sample (Mishra and Passos, 2021; Li et al., 2023). Essentially, 1DCNN is a type of neural network that utilizes convolutional layers to extract features from spectral data, which is treated as a one-dimensional sequence of data points, followed by fully connected layers to make precise predictions. By applying convolutional layers along the spectral axis, 1DCNN can capture spectral correlations and dependencies, thereby extracting relevant and discriminative features for predicting chemical composition content.

**Figure 1:**
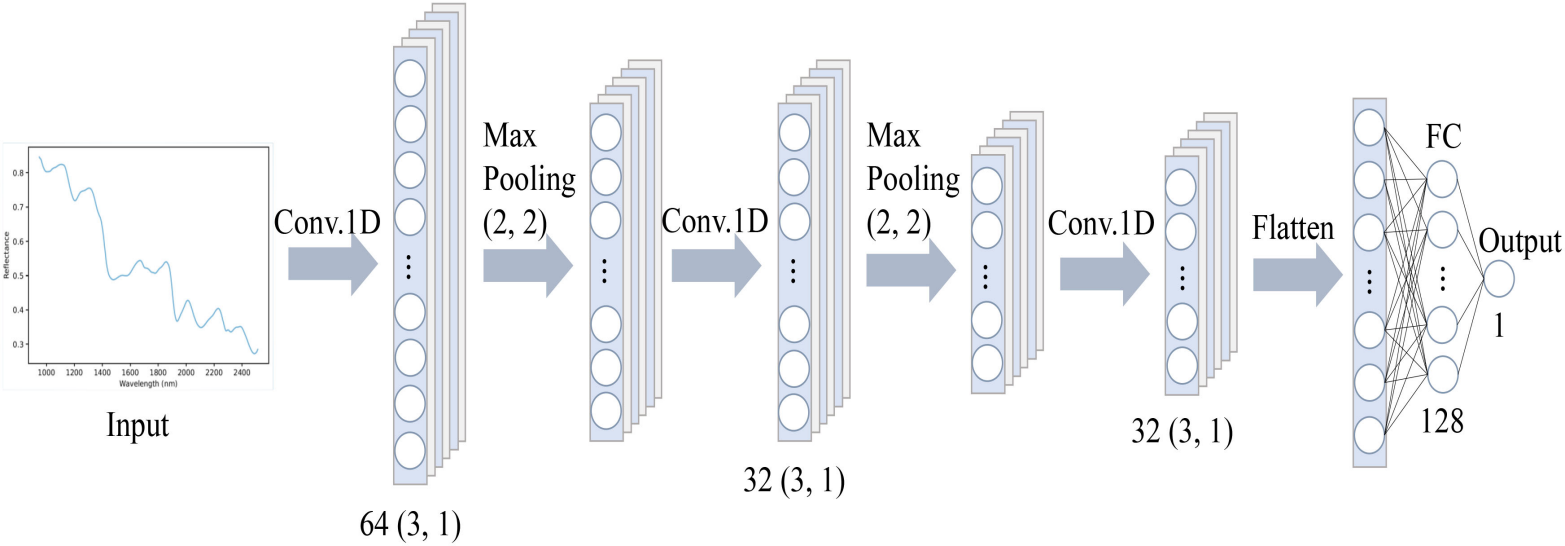
The CNN architecture utilized for predicting the levels of isoflavones and starch in PTR.

The 1DCNN architecture offers a notable advantage in handling large amounts of spectral data, making it well-suited for HSI applications with numerous spectral bands per pixel. This capability enables efficient processing of extensive datasets, resulting in highly accurate predictions. In this study, we employed a 1DCNN framework to predict the concentrations of puerarin, puerarin apioside, daidzin, daidzein, and starch in PTR, as shown in **Figure 1**. The designed CNN architecture comprises an input layer, three convolutional layers, two maxpooling layers, one fully connected layer, and an output layer. To be specific, the convolutional layers utilize a kernel size of 3×1, with 64, 32, and 32 kernels, respectively.

### 2.6 Performance evaluation metrics

This study employed several evaluation metrics to assess the accuracy and reliability of the models, including root mean squared error (RMSE), mean absolute error (MAE), coefficient of determination (R^2^) and residual predictive deviation (RPD).

Assuming that the predicted values and actual values are denoted by vectors $\hat{y}=\left[{\hat{y}}_{1},{\hat{y}}_{2},\ldots ,{\hat{y}}_{n}\right]$ and $y=\left[y_{1},y_{2},\ldots ,y_{n}\right]$ respectively, the formula for calculating the performance metric are as follows.


(3) $$\begin{array}{l} \text{RMSE}=\sqrt{\frac{1}{n}\sum_{i=1}^{n}{\left(y_{\text{i}}-{\hat{y}}_{\text{i}}\right)}^{2}},\in \left[0,+\infty \right)\\ \text{MAE}=\frac{1}{n}\sum_{i=1}^{n}\left|{\hat{y}}_{\text{i}}-y_{\text{i}}\right|\\ R^{2}=1-\frac{\sum_{i=1}^{n}{\left(y_{i}-{\hat{y}}_{i}\right)}^{2}}{\sum_{i=1}^{n}{\left(y_{i}-\bar{y}\right)}^{2}},\in \left[1,0\right]\\ \text{RPD}=\frac{\sqrt{\frac{1}{n-1}\sum_{i=1}^{n}{\left(y_{i}-\bar{y}\right)}^{2}}}{\sqrt{\frac{1}{n}\sum_{i=1}^{n}{\left(y_{i}-{\hat{y}}_{i}\right)}^{2}}} \end{array}$$

where *n* denotes the sample size, ${\hat{y}}_{i}$ represents the predicted value of the *i^th^* element, *y_i_* represents the actual value of the *i^th^* element, and $\bar{y}$ is the mean value.

When evaluating the performance of different models, the one that with the lowest RMSE is generally regarded as having better predictive accuracy. A lower MAE value suggests that the model has smaller prediction errors, while a higher RPD value indicates a better predictive accuracy compared to the reference data. Additionally, the model with the highest coefficient of determination indicates a stronger linear relationship between predicted and actual values, which is also an important factor to consider when comparing model performance.

### 2.7 Software tools and configurations

The correction and visualization of hyperspectral image data were carried out in the Environment for Visualizing Images (ENVI) 5.3 software from ITT Visual Information Solutions, Inc. in Boulder, CO, USA. The experiments for the proposed DL models were conducted on a server equipped with an Intel(R) Xeon(R) Platinum 8368 CPU @ 2.40GHz (251G RAM) and a GA100 graphics card (A100 PCIe 80GB GPU), running the Ubuntu Linux 21.04 operating system. The model’s compilation was created in the Python programming language (Python 3.7.10) and implemented using TensorFlow 2.2.0 and CUDA 11.7. During the network training, the cross-entropy loss function and the Adam optimization algorithm were utilized, while the learning rate, and batch size were set to 0.001 and 16, respectively. The dataset was randomly split into a training set (80% of the dataset) and a test set (20% of the dataset). To ensure the reliability of the results, all experiments were conducted and averaged over 10 independent runs.

## 3 Results and discussion

### 3.1 Content of isoflavones and starch in PTR

The contents of isoflavones and starch in PTR samples were determined and presented in **Table 1**. Among the total samples, starch had the highest mean concentration of 506.71 mg/g with a standard deviation of 29.08 mg/g, while daidzein had the lowest mean concentration of 0.2901 mg/g with a standard deviation of 0.07 mg/kg. In general, the trends in the concentrations of isoflavones and starch were similar across most samples. Nonetheless, the results indicate that the starch content in PTR samples remains relatively constant.

**Table 1:** Content of isoflavones and starch in PTR.

Nutrient		Content detection indexes		
		Mean(mg/g)	Max(mg/g)	Min(mg/g)
Isoflavones	Puerarin Puerarin apioside	2.2863±0.74 0.4212±0.12	4.1164 0.8053	0.8321 0.1820
	Daidzin	0.3465±0.11	0.6271	0.1285
	Daidzein	0.2091±0.07	0.5878	0.1155
Starch		506.71±29.08	615.12	448.94

### 3.2 Spectral characteristics of PTR

**Figure 2:**
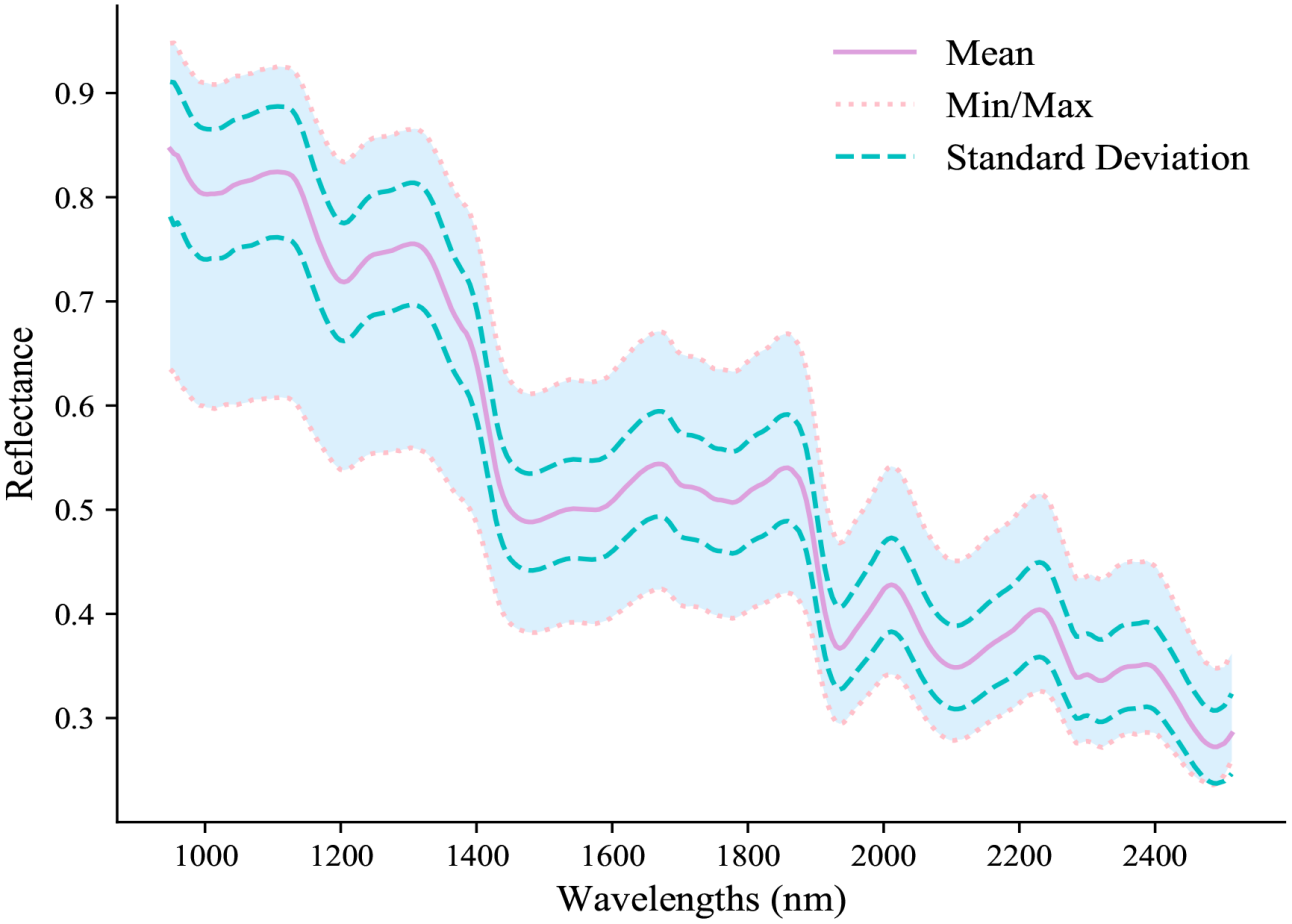
The mean, minimum, maximum and standard deviation spectral reflectance for PTR.

The spectral reflectance of PTR samples was analyzed over the range of 948.72−2512.97 nm, and the mean, minimum, maximum, and standard deviation values are depicted in **Figure 2**. The observed spectral reflectance exhibited a decrease with increasing wavelength, which is consistent with the typical optical properties of PTR. The spectral profile displayed multiple peaks and valleys corresponding to absorptions, where the primary absorption bands were appeared at 990 nm, 1200 nm, 1450 nm, 1550 nm, 1765 nm, 1942 nm, 2112 nm, and 2278 nm. Specifically, the absorption peaks around 990 nm and 1450 nm can be attributed to the third overtone of O-H stretching in alcohol or phenol-OH and the first overtone stretching of O-H in water (Barbin et al., 2013; Zhang et al., 2019). Similarly, the absorption peak around 1200 nm primarily arises from the second overtone of C-H stretching in starch (He et al., 2013). Moreover, the absorption peaks at 1550 nm and around 1765 nm correspond to the first overtone stretching and the third overtone stretching of N-H (Lü et al., 2017), respectively. Additionally, the absorption peak around 2278 nm mainly results from the first overtone stretching of C-H in aliphatic compounds such as fatty acids (He et al., 2017). Furthermore, the absorption peaks around 1942 nm and 2112 nm mainly attributed to the characteristic absorption of N-H and -NH_2_ groups, which are associated with the presence of proteins and amino acids in PTR (Wang et al., 2013; Qiu et al., 2021). Therefore, the obtained spectral profile provides a solid foundation for qualitative analysis of various PTR attributes using chemometrics and hyperspectral techniques.

However, the intricate composition of PTR samples presents a challenge for the direct quantification of isoflavones and starch levels using wavelengths from the spectral curve. Thus, further research is crucial to validate these observations and construct accurate and reliable models that can predict the concentrations of isoflavones and starch based on spectral data. In this regard, chemometric approaches were employed to explore the correlation between spectral reflectance and the levels of isoflavone and starch in the samples. This will help overcome the limitations of conventional analytical techniques and enhance the efficiency and accuracy of quality control in the food industry.

### 3.3 Prediction model’s performance when considering full wavelengths

#### 3.3.1 Conventional prediction models

Firstly, we conducted a comparison of three models: SVR, PLSR, and CatBoost. In order to enhance the prediction performance, various preprocessing methods were evaluated, including SNV, MSC, SG, FD, and SD, as discussed in section 2.5.1. The prediction results of the model combinations are summarized in **Supplementary Table 1** (see **Supplementary materials**). When comparing the results with the original spectral data, it was observed that the preprocessing methods led to varying degrees of improvement in predictive performance. Among these preprocessing methods, SG exhibited superior performance in predicting the contents of isoflavones and starch.

Regarding the prediction of isoflavone and starch contents, all three models demonstrated good performance, with most of the R^2^ exceeding 0.8 and the RPD values greater than 2.5. These results indicate that the combination of HSI technology and data analysis methods is feasible for determining the levels of puerarin, puerarin apioside, daidzin, daidzein, and starch in PTR. It is worth noting that the prediction performance of the three models, combined with all the preprocessing methods, was highest for the starch content compared to other components. The average values of R^2^, RPD, RMSE, and MAE for starch were found to be 0.8527, 2.6258, 11.7018, and 8.3385, respectively.

**Figure 3:**
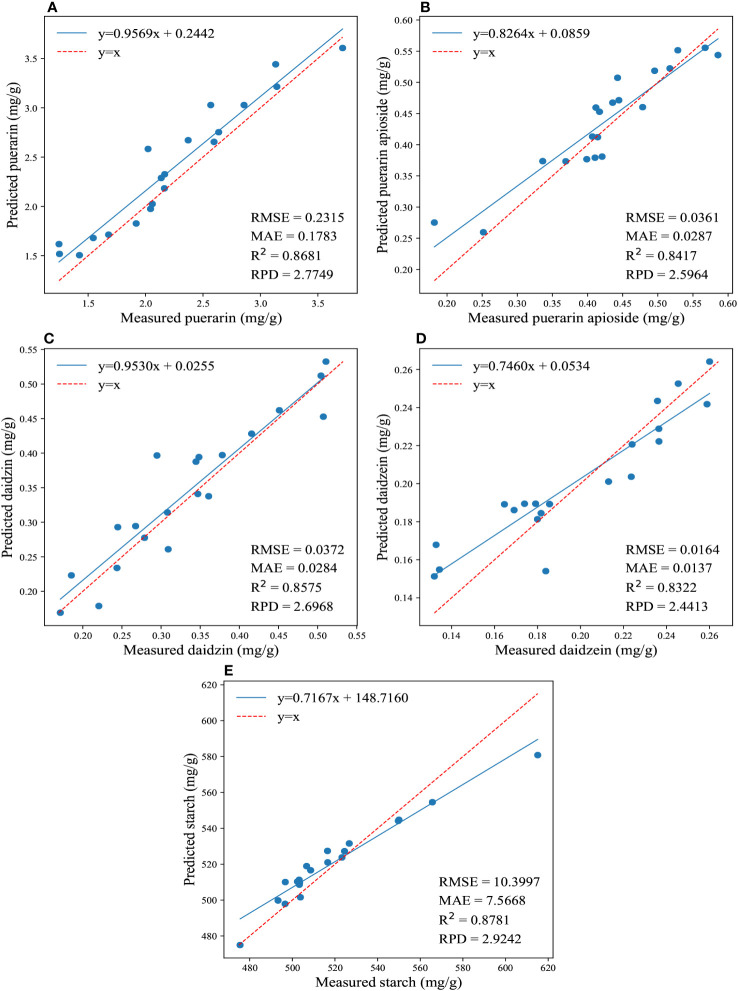
The regression results for the reference and predicted values of the four isoflavones and starch contents are depicted in **(A-E)**, which illustrate the predictions of puerarin, puerarin apioside, daidzin, daidzein, and starch contents using the full wavelengths and conventional models.

Furthermore, **Figure 3** illustrates the regression results for the reference and predicted values of the four isoflavones and starch contents obtained by applying the traditional model using full wavelengths. It showcases the best performance achieved by this model. The PLSR yielded better predictions for the content of daidzein and starch, resulting in the lowest values of RMSE (0.01645, 10.3977) and MAE (0.01366, 7.5668), as well as the highest RPD (2.4413, 2.9242) and R^2^ (0.8322, 0.8781). On the other hand, CatBoost exhibited the best prediction accuracy for puerarin, puerarin apioside, and daidzin contents, with the lowest values of RMSE (0.2315, 0.036, 0.0372) and MAE (0.1783, 0.0287, 0.0284), as well as the highest RPD (2.7749, 2.5964, 2.6968) and R^2^ (0.8681, 0.8417, 0.8575).

Overall, the combination of HSI and traditional machine learning techniques has proven to be a valuable approach for predicting the levels of isoflavones and starch in PTR. Among the findings of the experiment, CatBoost demonstrated superior performance compared to the other investigated models. Nevertheless, there is still room for further improvement in the predictive accuracy of these models. One promising direction for enhancement is the incorporation of advanced wavelength selection methods.

#### 3.3.2 Deep learning prediction model

In subsection 3.3.1, it has been demonstrated that the SG yields the best preprocessing results. Therefore, in following experiment, only the SG method was employed as the preprocessing technique. The outcomes of predicting the isoflavones and starch contents based on full wavelengths using the 1DCNN model are presented in **Table 2**. The results clearly indicate that the 1DCNN model outperformed the previously compared traditional models. The preprocessed data were directly fed into the 1DCNN, resulting in average R^2^ values above 0.90 and RPD values exceeding 3.20 for the five different components analyzed in PTR.

**Table 2:** The prediction results of the content of puerarin, puerarin apioside, daidzin, daidzein, and starch in PTR using 1DCNN with full wavelengths.

Models	Chemical indexes	Evaluation Metrics			
		R^2^	RMSE	MAE	RPD
SG + 1DCNN	Puerarin Puerarin apioside Daidzin Daidzein	0.9183 0.8955 0.9014 0.8882	0.2051 0.0349 0.0309 0.0123	0.1547 0.0274 0.0278 0.0093	3.4975 3.0939 3.1847 2.9910
	Starch	0.9054	7.8528	6.4348	3.2512

In terms of predicting puerarin, the 1DCNN model demonstrated improved performance compared to the traditional models, with an average increase of 0.0546 and 0.7804 in R^2^ and RPD, respectively. Similarly, for puerarin apioside, the 1DCNN model surpassed the other models, yielding an average increase of 0.0696 and 0.6653 in R^2^ and RPD, respectively. Furthermore, the 1DCNN model exhibited enhanced prediction results for daidzin and daidzein, resulting in average increases of 0.0566, 0.0673 and 0.6226, 0.6252 in R^2^ and RPD, respectively. Likewise, the 1DCNN model showcased superior performance in predicting starch, with average increases of 0.0359 and 0.4598 in R^2^ and RPD, respectively.

**Figure 4:**
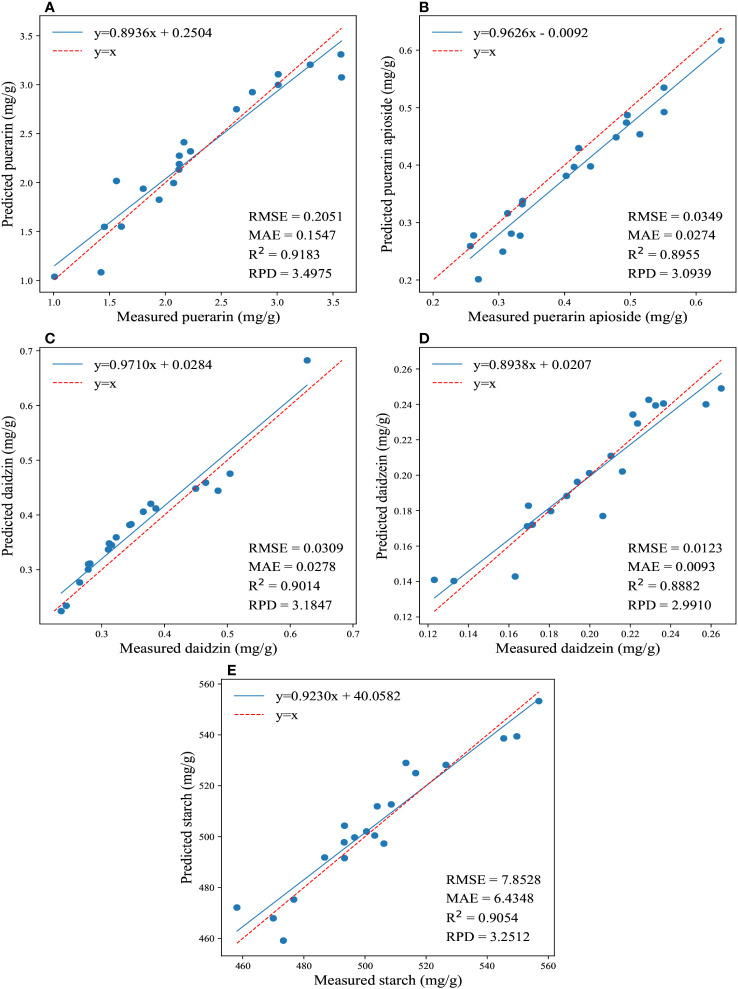
The regression results for the reference and predicted values of the four isoflavones and starch contents are depicted in **(A-E)**, which illustrate the predictions of puerarin, puerarin apioside, daidzin, daidzein, and starch contents using the full wavelengths and 1DCNN.

The regression results for the reference and predicted values of the four isoflavones and starch contents are shown in **Figure 4**. Overall, the 1DCNN model displayed superior and consistent performance in predicting the contents of puerarin and starch in PTR. Remarkably, notwithstanding without very complex design, the 1DCNN model outperformed traditional algorithms, highlighting the superiority of DL algorithms. Moreover, it should be emphasized that prediction results can be influenced by different parameter settings. In our study, we set the epoch to 200 for predicting the concentrations of the four isoflavones, and 1000 for determining the starch content. These findings suggest that utilizing a CNN model with spectral data is a feasible and effective approach for predicting the isoflavones and starch contents in PTR root.

### 3.4 Prediction model’s performance when considering effective wavelengths

In this section, three methods, namely SPA, CARS, and UVE, were employed to select effective wavelengths, aiming to improve the prediction performance of the models. For the prediction of puerarin, the three methods selected 14, 19, and 25 important variables from the full range of wavelengths. Similarly, for the prediction of puerarin apioside, 10, 17, and 22 significant wavelengths were chosen. For daidzin and daidzein, SPA, CARS, and UVE selected 16, 31, and 33, as well as 12, 23, and 28 important variables, respectively, from a pool of 288 variables. Lastly, 13, 28, and 30 significant wavelengths were chosen for starch content prediction.

**Figure 5:**
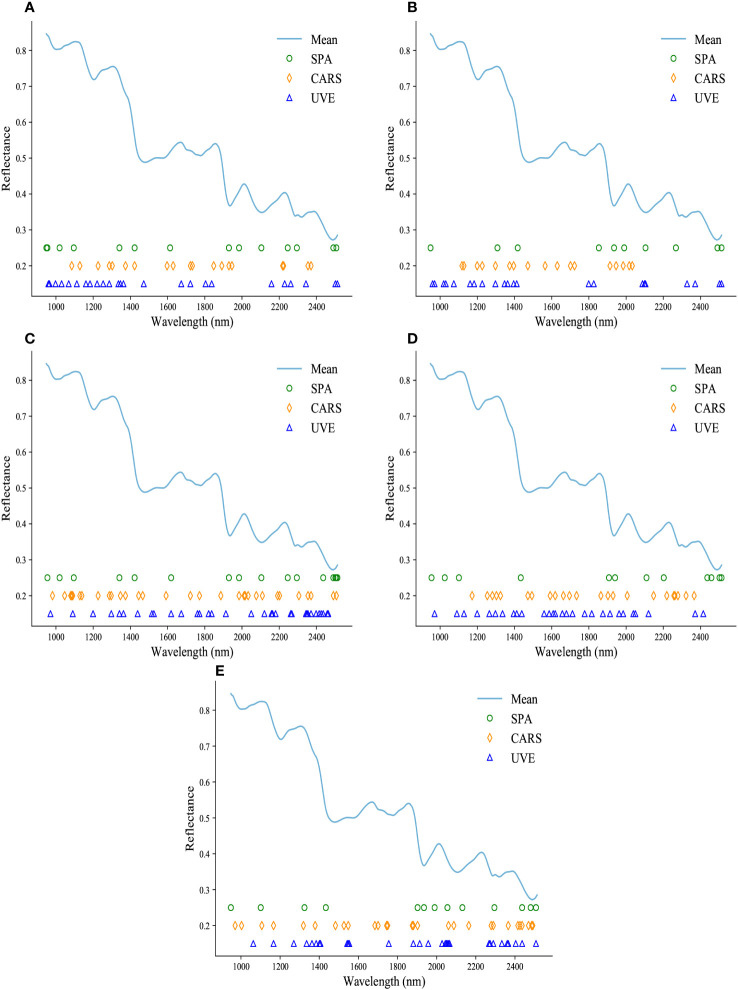
The specific locations of important wavelengths extracted by SPA, CARS, and UVE are presented in **(A-E)**, showcasing the prediction of puerarin, puerarin apioside, daidzin, daidzein, and starch contents.

The specific distribution of the selected feature wavelengths for each component prediction is shown in **Figure 5**. It is evident that despite the distinct principles guiding the three wavelength selection methods, they consistently converge on similar significant wavelength ranges. These selected wavelengths are primarily concentrated within regions that exhibit prominent and representative features, aligning with the absorption bands illustrated in **Figure 2**. As depicted in **Figure 2**, which are known to be highly correlated with the respective chemical constituents. This consistency in wavelength selection reinforces the notion that these specific regions of the spectrum contain valuable information that is closely linked to the chemical composition being analyzed.

**Supplementary Table 2** (see **Supplementary materials**) presents the prediction results of these algorithms for the three traditional models. From the results in **Supplementary Table 2**, it is evident that all three band selection methods improved the prediction ability of the traditional models. Notably, SPA exhibited the most substantial improvement, surpassing CARS and UVE.

**Figure 6:**
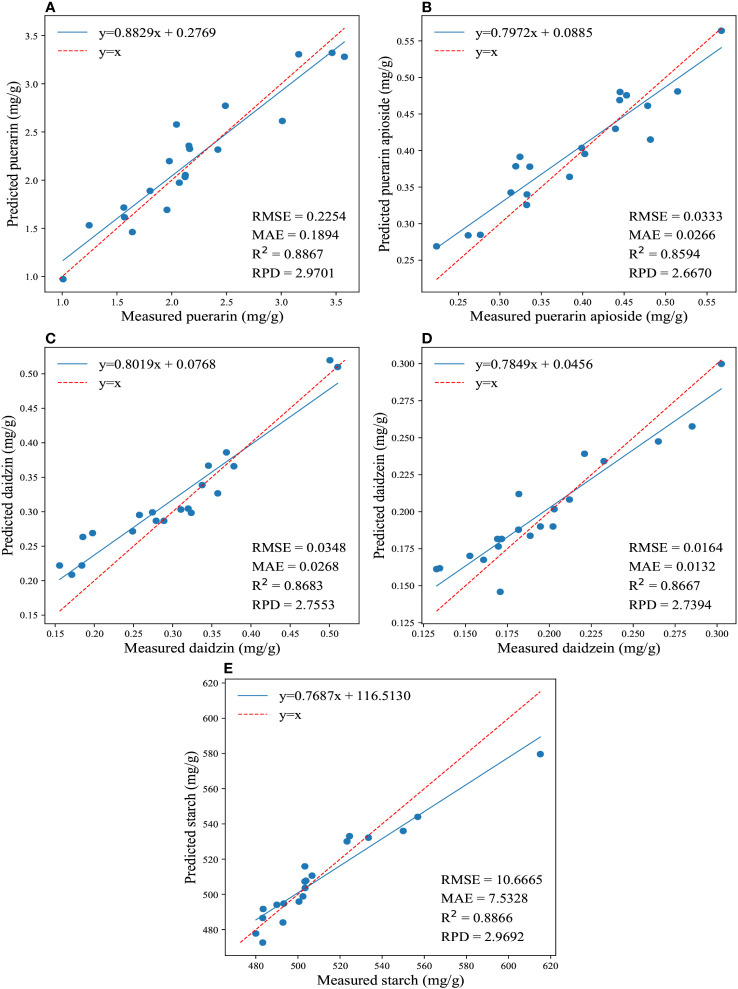
The regression results for the reference and predicted values of the four isoflavones and starch contents are depicted in **(A-E)**, which illustrate the predictions of puerarin, puerarin apioside, daidzin, daidzein, and starch contents using the effective wavelengths and conventional models.

In addition, **Figure 6** illustrates the regression results for the reference and predicted values of the four isoflavones and starch contents by applying the aforementioned three traditional model, showcasing the best performance achieved. For the prediction of puerarin, puerarin apioside, daidzin, and daidzein, the SG-SPA-CatBoost combination demonstrated the best performance among the conventional algorithms. The average values of the R^2^, RPD, RMSE, and MAE were determined to be 0.8703, 2.783, 0.0775, and 0.0640, respectively. Conversely, for starch prediction, SG-SPA-PLSR exhibited the highest prediction accuracy, with the lowest RMSE (10.667) and MAE (7.533), as well as the highest RPD (2.9692) and R^2^ (0.8876).

**Figure 7:**
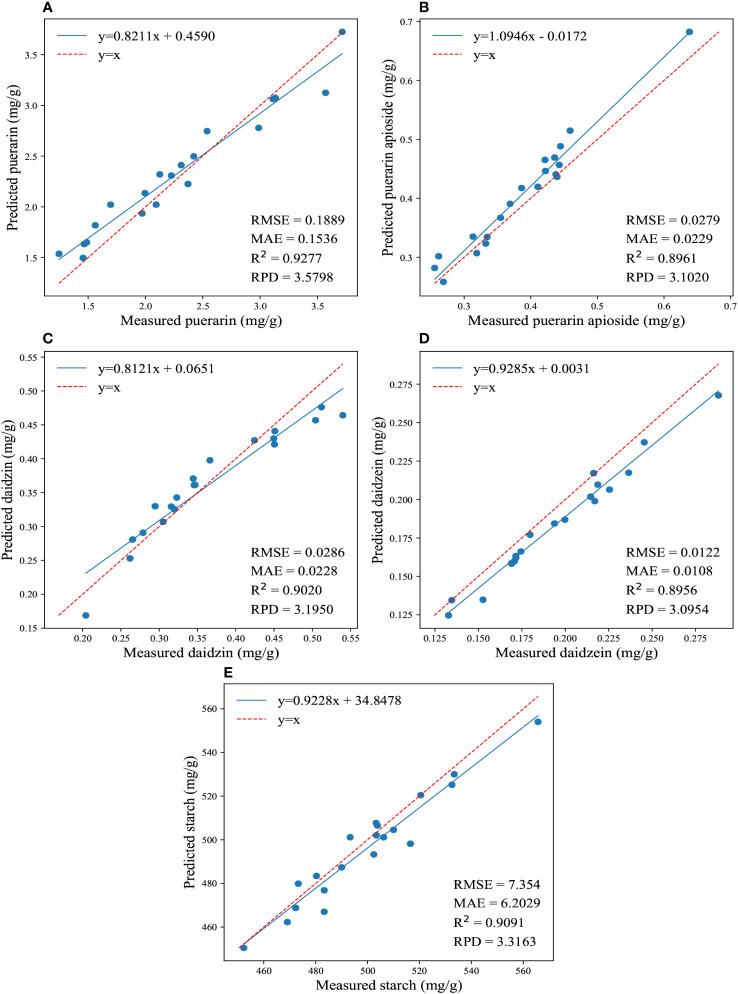
The regression results for the reference and predicted values of the four isoflavones and starch contents are displayed in **(A-E)**, illustrating the prediction of puerarin, puerarin apioside, daidzin, daidzein, and starch contents using the effective wavelengths and 1DCNN.

**Table 3** displays the prediction results of the effective wavelengths and CNN model, while **Figure 7** illustrates the regression results for the reference and predicted values of the four isoflavones and starch contents by utilizing the effective wavelengths and 1DCNN. However, it is important to highlight that in the case of the CNN model, the inclusion of effective wavelength selection did not result in a significant improvement in performance, and in some cases, even led to a slight decrease compared to the model using full wavelengths. Nevertheless, it remains evident that DL models are less susceptible to the impact of different wavelength selection methods, and the CNN model utilizing full wavelengths consistently demonstrated superior performance. This underscores the inherent strength of DL in effectively handling intricate and highly complex data. By virtue of the end-to-end training process, DL models autonomously extract non-linear hidden features from samples in a globally optimized manner. It is this interconnected process that explains the underlying reason for the observed decrease in performance of the CNN model when wavelength selection is employed.

**Table 3:** The prediction results of the content of puerarin, puerarin apioside, daidzin, daidzein, and starch in PTR using 1DCNN with effective wavelengths.

Models	Chemical indexes	Method	Number of bands		Evaluation Metrics		
				R^2^	RMSE	MAE	RPD
SG + 1DCNN	Puerarin Puerarin apioside Daidzin	SPA CARS UVE SPA CARS UVE SPA CARS UVE	14 19 25 10 17 22 16 31 33	0.9277 0.9220 0.9129 0.8961 0.8918 0.8903 0.9020 0.9014 0.8992	0.1889 0.2012 0.2212 0.0279 0.0313 0.0328 0.0286 0.0315 0.0324	0.1536 0.1670 0.1838 0.0229 0.0249 0.0273 0.0228 0.0245 0.0267	3.7199 3.5798 3.3876 3.1020 3.0407 3.0187 3.1950 3.1844 3.1495
	Daidzein	SPA CARS UVE	12 23 28	0.8956 0.8851 0.8833	0.0122 0.0137 0.0159	0.0108 0.0118 0.0136	3.0954 2.9502 2.9277
	Starch	SPA CARS UVE	13 28 30	0.9091 0.8977 0.8892	7.7354 8.0546 8.7960	6.2029 7.1920 7.6628	3.3163 3.1271 3.0038

### 3.5 Discussion

The constituents and their corresponding concentrations are quintessential to determine the intrinsic quality and commercial value of a material. In this study, we employed a combination of HSI and data analysis techniques to accurately predict the content of key compounds in PTR. Through a comprehensive analysis of the results, it was evident that the integration of HSI with a deep learning model achieved satisfactory predictive performance. Given its convenience, non-destructiveness, and high-efficiency, this approach could serve as a viable and beneficial alternative to complicated physical and chemical procedures. It could be implemented in off‐line rapid analysis or on‐line quality control for quality evaluation of PTR.

First, among the traditional algorithms compared in this study, the performance of PLSR algorithm was found to be relatively stable and moderate overall, which is consistent with its frequent utilization in similar studies involving hyperspectral prediction (Aredo et al., 2017; Hu et al., 2021). However, Catboost algorithm, despite being less commonly used in hyperspectral analysis, emerged as a robust competitor in our study. It outperformed PLSR and SVR in predicting certain components and exhibited slightly superior overall performance compared to PLSR and SVR. This can be attributed to CatBoost’s unique characteristics, including the utilization of ensemble models, embedded regularization, and automatic handling of categorical features. These features contribute to enhanced learning capability, improved generalization, and increased robustness of the model (Hancock and Khoshgoftaar, 2020). Consequently, CatBoost exhibits better performance in predicting hyperspectral data compared to PLSR and SVR, particularly in scenarios with limited sample sizes. Unsurprisingly, the presence of redundant spectral bands significantly impacts the performance of the algorithms, presenting a notable challenge for traditional methods. In the comparison of various band selection algorithms (SPA, CARS, VUE), it was determined that SPA yielded the best results. Introducing the SPA significantly improved the algorithm’s performance, as observed in numerous similar studies (Zhang et al., 2017; Zhu et al., 2017).

In contrast, our study demonstrates that deep learning methods exhibit superior and robust predictive performance. The average R^2^ for component prediction reaches 0.9, even without the need for wavelength selection. This remarkable performance can be attributed to the autonomous learning capability of deep learning models, which effectively capture intricate mapping relationships between spectra and components, unaffected by spectral redundancy and noise. These findings are consistent with previous research studies (Dargan et al., 2020; Zhang C. et al., 2020; Wang et al., 2021). Interestingly, our investigation reveals that the introduction of wavelength selection does not significantly enhance the performance. This observation may be attributed to the distinct approach of CNN as end-to-end learning models, which differ in learning feature representations compared to traditional methods. Consequently, the feature subset selected for traditional models may have limited impact on CNNs, leading to negligible performance improvements. Moreover, it is worth mentioning that the 1DCNN used in our study was relatively simple, incorporating commonly used convolution and pooling modules. Recent advancements in deep learning have introduced innovative techniques, particularly attention mechanisms, which enable models to differentially process various features (Sun et al., 2019). By assigning greater weights to key features, attention mechanisms enhance the influence of crucial features, thereby facilitating more accurate judgments and predictions in spectral classification and prediction tasks (Zhang et al., 2022a; Wang Y. et al., 2023). Therefore, the utilization of more complex deep networks and mechanisms, such as attention, holds potential for further improving prediction performance.

Furthermore, although CNN have shown remarkable success in various component prediction tasks (Dargan et al., 2020; Zhang C. et al., 2020; Wang et al., 2021; Zhang et al., 2022a), including the findings of this study, they are not without limitations. One such limitation is the challenge in interpreting the learning process of CNNs. The interpretability of deep learning can be crucial in understanding and explaining the importance of specific spectral bands (Zhang L. et al., 2020). Furthermore, to fully harness the power of deep learning models, a larger quantity of collected sample data is expected. Currently, some studies have started exploring the utilization of generative adversarial networks (GANs) for generating reliable synthetic spectral data as a form of data augmentation (Wang et al., 2019; Zhang et al., 2022b). This technique has demonstrated the potential to further enhance the performance of CNNs in spectral prediction tasks.

Finally, with the deepening comprehension of the intrinsic mechanisms of CNNs, research combining CNNs and spectroscopy for food quality evaluation has been gaining momentum. These studies indicate that CNNs still hold strong potential even in small sample scenarios and, in many cases, outperform traditional modeling methods (Zhang C. et al., 2020; Zhang et al., 2021; Li et al., 2023), consistent with the findings of this study. Therefore, further development of the combination of CNNs and spectroscopy, leveraging the strengths of CNNs in processing images and high-dimensional data, is expected to bring new advances in fields such as food analysis and quality assessment. However, it is important to address and mitigate the drawbacks and limitations of CNNs, employing targeted strategies in their application.

## 4 Conclusions

The accurate detection of isoflavones and starch content in PTR is of paramount importance in ensuring its quality and safety. In this study, we pioneered a noninvasive, efficient detection method using HSI and machine learning algorithms to predict puerarin, puerarin apioside, daidzin, daidzein, and starch content in PTR. By employing various spectral preprocessing techniques and wavelength selection methods, we compared several traditional machine learning algorithms (PLS, SVM, CatBoost) with a 1DCNN model. Our results unequivocally demonstrated that the combination of HSI and 1DCNN yielded superior predictive performance, with an average R^2^ exceeding 0.9 for all components. Importantly, unlike traditional methods, the performance of the 1DCNN model was not significantly dependent on feature wavelength selection.

Overall, our findings highlight the tremendous potential of HSI coupled with a deep learning model in simultaneously determining multiple key components in PTR. Considering the advantages of spectral detection, this approach offers a viable and efficient solution for non-destructive quality control assessment of PTR, which can be applicable to both off-line and on-line settings. Furthermore, future research can delve into advanced deep learning techniques like attention mechanisms to boost predictive capabilities and broaden HSI applications in quality assessment.

## Data availability statement

The raw data supporting the conclusions of this article will be made available by the authors, without undue reservation.

## Author contributions

HH: Data curation, Writing – original draft. TW: Investigation, Writing – review & editing. YW: Data curation, Writing – review & editing. ZX: Methodology, Writing – review & editing. SC: Data curation, Writing – review & editing. LF: Data curation, Formal Analysis, Writing – review & editing. HX: Writing – review & editing. XM: Writing – review & editing. LH: Funding acquisition, Investigation, Writing – review & editing.

## Supplementary material

Supplementary Table 1 Prediction results of the content of puerarin, puerarin apioside, daidzin, daidzein, and starch in PTR by traditional models using full wavelengths.

Supplementary Table 2 Prediction results of the content of puerarin, puerarin apioside, daidzin, daidzein, and starch in PTR by using effective wavelengths and traditional algorithms.
