# Supplementary material for: Non-destructive prediction of isoflavone and starch by hyperspectral imaging and deep learning in Puerariae Thomsonii Radix
Source: Front Plant Sci. 2023 Oct 25;14:1271320. doi: 10.3389/fpls.2023.1271320 (PMC10634472; doi:10.3389/fpls.2023.1271320)
Supplement: Supplementary Table 1 — Prediction results of the content of puerarin, puerarin apioside, daidzin, daidzein, and starch in PTR by traditional models using full wavelengths. [file Table_1.pdf]

Table 1: Prediction results of the content of puerarin, puerarin apioside, daidzin, daidzein, and starch in PTR by traditional models using full wavelengths

Chemical indexes	Models	Preprocessing	Evaluation Metrics			
			R^2	RMSE	MAE	RPD
puerarin	SVR	Raw	0.8489	0.2602	0.2178	2.5723
		SNV	0.8496	0.2709	0.2110	2.5783
		MSC	0.8398	0.2719	0.1937	2.4983
		SG	0.8570	0.2290	0.1741	2.6447
		FD	0.8342	0.3024	0.2244	2.4556
		SD	0.8295	0.3204	0.2572	2.4215
	PLSR	Raw	0.8420	0.2709	0.2298	2.5161
		SNV	0.8283	0.2992	0.2452	2.4131
		MSC	0.8434	0.2858	0.2359	2.5670
		SG	0.8660	0.2576	0.2230	2.7316
		FD	0.8388	0.3095	0.2366	2.4909
		SD	0.8143	0.3263	0.2735	2.3207
	CatBoost	Raw	0.8551	0.2437	0.1966	2.6273
		SNV	0.8305	0.2616	0.1827	2.4287
		MSC	0.8549	0.2326	0.1876	2.6259
		SG	0.8681	0.2315	0.1783	2.7749
		FD	0.8428	0.2434	0.2003	2.5222
		SD	0.8336	0.2570	0.2090	2.4518
	SVR	Raw	0.8024	0.0505	0.0446	2.2494
		SNV	0.7999	0.0469	0.0372	2.2357
		MSC	0.7905	0.0473	0.0394	2.1848
		SG	0.8134	0.0462	0.0383	2.3148
		FD	0.7839	0.0530	0.0410	2.1516
		SD	0.7743	0.0534	0.0401	2.1048

Puerarin apioside

	PLSR	Raw	0.8181	0.0587	0.0468	2.3445
		SNV	0.8238	0.0566	0.0447	2.3823
		MSC	0.8271	0.0539	0.0413	2.4045
		SG	0.8226	0.0506	0.0393	2.3745
		FD	0.8188	0.0541	0.0402	2.3494
		SD	0.8021	0.0560	0.0476	2.2481
	CatBoost	Raw	0.8247	0.0593	0.0426	2.3885
		SNV	0.8335	0.0455	0.0373	2.4510
		MSC	0.8293	0.0426	0.0331	2.4201
		SG	0.8417	0.0361	0.0287	2.5964
		FD	0.8207	0.5240	0.3118	2.3616
		SD	0.8176	0.0602	0.4034	2.3417
	SVR	Raw	0.8023	0.0548	0.0471	2.2489
		SNV	0.8059	0.0479	0.0406	2.2697
		MSC	0.7892	0.0562	0.0497	2.1781
		SG	0.8247	0.0446	0.0380	2.3884
		FD	0.7947	0.0481	0.0396	2.2071
		SD	0.7867	0.5008	0.0420	2.1654
	PLSR	Raw	0.8413	0.0452	0.0372	2.5105
		SNV	0.8496	0.0453	0.0388	2.5789
daidzin		MSC	0.8288	0.0496	0.0429	2.4171
		SG	0.8522	0.0417	0.0351	2.6012
		FD	0.8339	0.0493	0.0408	2.4537
		SD	0.8182	0.0525	0.0419	2.3452
	CatBoost	Raw	0.8591	0.0373	0.0305	2.6640
		SNV	0.8573	0.0390	0.0299	2.6473
		MSC	0.8358	0.0435	0.0349	2.4681
		SG	0.8575	0.0372	0.0284	2.6968
		FD	0.8395	0.0459	0.0342	2.4962
		SD	0.8224	0.0484	0.0357	2.3727

	SVR	Raw	0.7838	0.0320	0.0278	2.1507
		SNV	0.7845	0.0316	0.0265	2.1541
		MSC	0.7575	0.0340	0.0292	2.0305
		SG	0.8091	0.0309	0.0276	2.2889
		FD	0.7403	0.0360	0.0286	1.9622
		SD	0.7281	0.0369	0.0321	1.9179
	PLSR	Raw	0.8152	0.0216	0.0173	2.3262
		SNV	0.8177	0.0201	0.0166	2.3422
		MSC	0.8055	0.0214	0.0187	2.2676
daidzein		SG	0.8322	0.0165	0.0137	2.4413
		FD	0.7968	0.0226	0.0184	2.2185
		SD	0.7836	0.0237	0.0197	2.1496
	CatBoost	Raw	0.8244	0.0242	0.0213	2.3861
		SNV	0.8253	0.0239	0.0167	2.3925
		MSC	0.8188	0.0268	0.0194	2.3495
		SG	0.8215	0.0187	0.0154	2.3672
		FD	0.7953	0.0286	0.0219	2.2104
		SD	0.7795	0.0310	0.0208	2.1297
	SVR	Raw	0.8240	12.9522	8.8712	2.3833
		SNV	0.8276	12.8816	8.8216	2.4086
		MSC	0.8286	12.7772	8.7499	2.4151
		SG	0.8599	11.2724	8.2151	2.6714
		FD	0.8294	12.3379	8.7192	2.4208
		SD	0.8244	12.9798	8.8514	2.3866
	PLSR	Raw	0.8664	10.8327	8.0296	2.7362
		SNV	0.8737	10.4673	7.6017	2.8136
		MSC	0.8688	10.8201	7.9563	2.7604
starch		SG	0.8781	10.3997	7.5668	2.9242
		FD	0.8589	11.4906	8.3171	2.6626
		SD	0.8592	11.6453	8.2022	2.6651

	CatBoost	Raw	0.8628	11.1838	8.4720	2.6996
		SNV	0.8655	11.1079	8.2804	2.7269
		MSC	0.8521	12.0579	8.4855	2.6005
		SG	0.8705	10.8085	7.9396	2.7786
		FD	0.8541	11.7377	8.4096	2.6183
		SD	0.8443	12.8798	8.6037	2.5341

Table 2: Prediction results of the content of puerarin, puerarin apioside, daidzin, daidzein, and starch in PTR by using effective wavelengths and traditional algorithms

Chemical indexes	Models	Method	Number of bands	Evaluation Metrics			
				R^2	RMSE	MAE	RPD
Puerarin	SVR	SPA	14	0.8672	0.2394	0.2009	2.7444
		CARS	19	0.8739	0.2472	0.1955	2.8158
		UVE	25	0.8653	0.2604	0.2192	2.7251
	PLSR	SPA	14	0.8714	0.2540	0.2105	2.7887
		CARS	19	0.8766	0.2463	0.2087	2.8465
		UVE	25	0.8664	0.2786	0.2424	2.7361
	CatBoost	SPA	14	0.8867	0.2254	0.1894	2.9701
		CARS	19	0.8761	0.2593	0.1789	2.8415
		UVE	25	0.8716	0.2433	0.2101	2.7911
Puerarin apioside	SVR	SPA	10	0.8267	0.0443	0.0341	2.4020
		CARS	17	0.8439	0.0430	0.0366	2.5309
		UVE	22	0.8254	0.0449	0.0374	2.3929
	PLSR	SPA	10	0.8424	0.0427	0.0315	2.5187
		CARS	17	0.8549	0.0355	0.0305	2.6253
		UVE	22	0.8486	0.0401	0.0354	2.5700
	CatBoost	SPA	10	0.8594	0.0333	0.0266	2.6670

		CARS	17	0.8565	0.0342	0.0274	2.6403
		UVE	22	0.8560	0.0367	0.0282	2.6348
	SVR	SPA	16	0.8278	0.0438	0.0363	2.4097
		CARS	31	0.8237	0.0475	0.0394	2.3815
		UVE	33	0.8221	0.0488	0.0424	2.3709
	PLSR	SPA	16	0.8557	0.0401	0.0331	2.6322
Daidzin		CARS	31	0.8571	0.0395	0.0320	2.6451
		UVE	33	0.8443	0.0403	0.0358	2.5340
	CatBoost	SPA	16	0.8683	0.0348	0.0268	2.7553
		CARS	31	0.8613	0.0393	0.0326	2.6848
		UVE	33	0.8621	0.0371	0.0289	2.6924
	SVR	SPA	12	0.8255	0.02125	0.0167	2.4025
		CARS	23	0.8168	0.02279	0.0194	2.3485
		UVE	28	0.8215	0.02155	0.0154	2.3662
	PLSR	SPA	12	0.8439	0.0196	0.0152	2.5309
Daidzein		CARS	23	0.8385	0.0204	0.0172	2.4885
		UVE	28	0.8304	0.0211	0.0176	2.4283
	CatBoost	SPA	12	0.8667	0.0164	0.0132	2.7394
		CARS	23	0.8635	0.0165	0.0127	2.7062
		UVE	28	0.8587	0.0179	0.0155	2.6599
	SVR	SPA	13	0.8750	11.5685	8.4755	2.8285
		CARS	28	0.8679	11.1625	9.2695	2.7509
		UVE	30	0.8676	11.9741	8.9439	2.7484
	PLSR	SPA	13	0.8866	10.6665	7.5328	2.9692
Starch		CARS	28	0.8812	11.1884	7.8486	2.9016
		UVE	30	0.8787	11.2586	8.6251	2.8713
	CatBoost	SPA	13	0.8762	8.0908	6.0178	2.8424
		CARS	28	0.8711	9.7795	6.0657	2.7848
		UVE	30	0.8627	11.1332	8.4156	2.6991
